# Increasing the Educational Value of the Orthopaedic Subinternship: The Design and Implementation of a Fourth-Year Medical Student Curriculum

**DOI:** 10.5435/JAAOSGlobal-D-20-00240

**Published:** 2021-01-19

**Authors:** Caitlin A. Orner, Sandeep P. Soin, Bilal Mahmood, John T. Gorczyca, Gregg T. Nicandri, Benedict F. DiGiovanni

**Affiliations:** From the Department of Orthopaedics and Rehabilitation, University of Rochester School of Medicine and Dentistry, Rochester, NY.

## Abstract

Most orthopaedic subinternships function as month-long interviews. These rotations remain relatively unstructured and lack standardization, and their overall educational value has been called into question. The goals of this educational initiative were to create a structured subinternship curriculum for orthopaedic applicants and to shift the focus of the subinternship from a month-long interview to an organized educational experience.

**Methods:**

After review of knowledge and skills expected for early orthopaedic residency under the structure of the Accreditation Council for Graduate Medical Education Milestones, a curriculum dedicated to orthopaedic subinternships was created. Students who completed the curriculum filled out anonymous Likert scale evaluations (rating their comfort/knowledge from 0 to 10 before and after their rotation) and answered open-ended qualitative questions.

**Results:**

Forty-six subinterns participated in the program over 3 years. Four weekly learning modules were designed and taught by orthopaedic residents, with faculty oversight of content and structure. Each monthly rotation began with an orthopaedic surgical skills laboratory and concluded with a case-based oral presentation. Weeks two and three covered different milestone-based topics and included didactic and skills development. Data analysis revealed that students reported notable improvement in knowledge and familiarity with each of the topics. The greatest improvements were in tibia intramedullary nailing and applying a tension band to an olecranon fracture. When asked which surgical skills station was the most helpful, 70% chose lag screw insertion and basic plating techniques. All students felt that creating their case presentation was productive.

**Conclusion:**

This educational initiative resulted in the successful design and implementation of a milestone-based orthopaedic surgery subinternship curriculum. The program was well received by students, contributed to learning and competency, and provided teaching opportunities for residents. The format and content of this subinternship curriculum can easily be adapted to regional and national teaching programs.

Sub-internships are a universal component of the fourth year of medical school. In 2017, O'Donnell et al^1^ found that orthopaedic surgery applicants spend an average of 15.5 weeks on orthopaedic rotations and complete an average of 2.4 away rotations. For a 12 month curriculum, this represents nearly 25% of the fourth of year medical school. Away rotations are expensive and require a notable time investment, and students must live for weeks at a time away from their home and family. It is clear, however, that participating in subinternships is an important part of the transition from medical student to orthopaedic resident because 56% of orthopaedic applicants match at a program where they rotated.^2^

Expectations of new orthopaedic residents are high. The Accreditation Council for Graduate Medical Education (ACGME) has published a set of orthopaedic surgery milestones to help track progress and guide curriculum development. According to the milestones, the knowledge and skill level expected of interns includes detailed patient workup and basic management decisions for multiple musculoskeletal conditions. Despite these high expectations, orthopaedic education remains underrepresented in the medical student curriculum. In 2016, DiGiovanni et al found that only 15% of American medical schools have required musculoskeletal rotations in the clinical years. On average, these rotations are only 2 weeks long.^3^ This highlights the importance of subinternships, which are a valuable opportunity for orthopaedic applicants to learn musculoskeletal knowledge and skills.

Currently, many subinternships remain relatively unstructured and lack standardization, and their overall educational value has been called into question.^1^ In addition, when subinternships function solely as “audition” rotations, the educational potential of the rotation is compromised. At our academic medical center, the orthopaedic surgery subinternship curriculum was redesigned and implemented in 2016 to include didactic and skills sessions designed specifically for rotating medical students. The objective of this initiative was to create an educationally rewarding, organized, and structured sub-internship, which we believe will better prepare future orthopaedic residents to treat patients and attain higher levels of competency.

## Methods

### Curriculum Design

At the University of Rochester, a subcommittee of educators and students was charged to develop specialty-specific competencies for rotating fourth-year students. This group combined the ACGME milestones, expert opinion, and evidence-based studies to develop specialty-specific competencies for multiple departments. In orthopaedics, these competencies were used to design and implement a weekly curriculum for fourth-year subinterns rotating at our institution.

The curriculum includes four weekly learning modules which were designed and taught by orthopaedic residents, with faculty oversight of content and structure. Sessions occurred Wednesday evenings from 5:30 pm to 7:30 pm. At our medical center, an orthopaedic surgery skills laboratory is located at the outpatient surgery center, which was the setting of the learning modules. Residents expressing an interest in medical student education were recruited to teach each topic, which includes preparing a didactic presentation and directing the skills session. Two residents are assigned to run each session, but all residents were invited to participate and help teach. On average, 3 to 4 residents were present at each weekly laboratory, with an approximately 1:2 ratio of residents to students.

A supply list was generated for each laboratory session (See Appendix 1, http://links.lww.com/JG9/A109) and was provided to the skills laboratory coordinator who assisted with setup. If implants were required, teaching sets were generously provided through an educational stipend with an industry partner.

The following is a summary of the 4-week curriculum:

Week 1: Each monthly rotation began with a general surgical skills laboratory. This session evolved based on student feedback to cover different topics. Students spent approximately 40 minutes at each of three stations including suturing/knot tying, external fixation, and basic drilling/plate application using sawbones.
Weeks 2 and 3: Weeks 2 and 3 focused on different ACGME milestone-based topics, with both didactic overview (30 minutes) and skills practice (90 minutes). The topics varied by month for each of the three months, and covered commonly encountered adult and pediatric fractures. Topics covered in this curriculum were distal radius fractures with reduction/splinting; ankle fractures with reduction/splinting; tibia fractures with intramedullary nailing; olecranon fractures with plating/tension band; hip fractures with dynamic hip screw and cephalomedullary nail; and acute fracture stabilization covering advanced splints (coaptation, ulnar/radial gutter, etc).
Week 4: Students concluded their rotation with a case based oral presentation. Students were asked to choose one interesting case in which they were involved, and they were challenged to develop a 10 minute oral case presentation and topic overview. All orthopaedic residents and faculty were invited to view the presentations, which were scheduled to occur in the evening.

**Figure 1:**
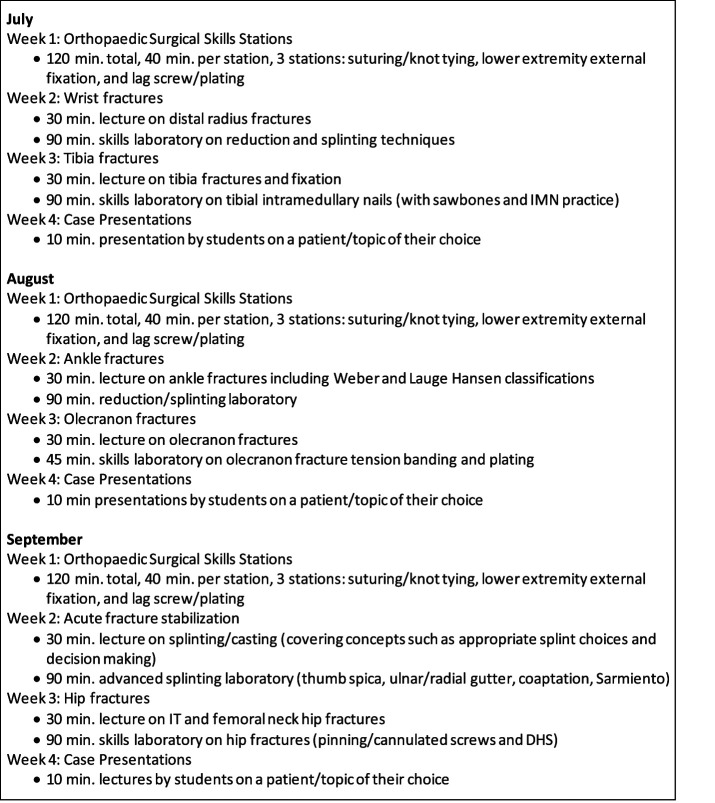
Illustration showing the sample subinternship curriculum.

See Figure 1 for an example of the curriculum.

### Evaluations

Students who completed the curriculum between 2016 and 2018 were asked to voluntarily fill out anonymous evaluations at the end of their rotation using a Likert scale to evaluate their comfort with skills before and after each module (10 = very comfortable). Students received different evaluations depending on which module they participated in. They also answered open-ended questions about their experience. Feedback from evaluations was used in curriculum development to refine the structure and content of the educational material. Permission to publish feedback from the anonymous evaluations was obtained from the University of Rochester IRB (study considered Exempt).

### Statistics

Microsoft Excel was used to calculate averages/standard deviations and T-tests for the Likert scale ratings on the student evaluations.

## Results

**Figure 2:**
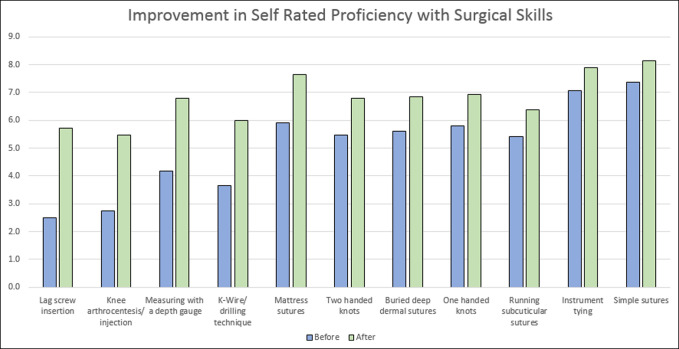
The chart illustrates a selection of skills/topics from Table 1 covered in the subintern curriculum. Students were asked to rate their comfort level (0 to 10 on a Likert scale) with these skills before and after their orthopedic rotation (Y axis).

Forty-six fourth-year medical students rotated over three academic years (2016 to 2018) during the months of July, August, and September. Orthopedic subinterns spend 4 weeks at our institution, and they rotate through 2 different subspecialties (including orthopaedic trauma, spine, adult reconstruction, foot/ankle, hand, sports, and pediatric orthopaedics). It is important to note that as feedback was collected from students, changes were made to the content of the program and to the evaluation forms; because students participated in different sessions, the number of responses was more limited for certain topics (see N column in Tables 1 and 2). Following the week 1 surgical skills laboratory, the students were asked to fill out evaluations rating their comfort level with surgical skills before and after the laboratory. Forty of 46 students (87%) returned this survey. These skills are listed in Table 1, along with student's self-rated improvement before and after the laboratory. Figure 2 shows that the skills students felt most improvement in were the more advanced skills specific to orthopaedic surgery, including lag screw insertion (∆ = 3.2 *P* < 0.05), knee arthrocentesis/injection (∆ = 2.7, *P* < 0.05), measuring with a depth gauge (∆ = 2.6, *P* < 0.05), and Kirschner wire/drilling technique (∆ = 2.3, *P* < 0.05).

**Table 1:** Column 1 Lists all Topics Covered in the Surgical Skills Laboratory During Week 1 of the Subintern Curriculum, Which the Students Were Asked to Rate Their Comfort Level With Before and After the Laboratory (0 to 10 on a Likert Scale)

	Before Rotation (Mean)	After Rotation (Mean)	∆ = After − Before (Mean)	N
Lag screw insertion	2.5	5.7	3.2^a^	40
Knee arthrocentesis/injection	2.8	5.5	2.7^a^	40
Measuring with a depth gauge	4.2	6.8	2.6^a^	40
Kirschner Wire technique/drilling technique	3.7	6.0	2.3^a^	15
Mattress sutures	5.9	7.6	1.7^a^	25
Two handed knots	5.5	6.8	1.3^a^	15
Buried deep dermal sutures	5.6	6.9	1.3^a^	40
One-handed knots	5.8	6.9	1.1^a^	15
Running subcuticular sutures	5.4	6.4	1.0^a^	40
Instrument tying	7.1	7.9	0.8^a^	40
Simple sutures	7.4	8.2	0.8^a^	40

N = number of student responses.

a Statistically significant (*P* < 0.05).

**Table 2:** Column 1 Lists all Topics Covered at Some Point the Sub-intern Curriculum, Which the Students Were Asked to Rate Their Comfort Level (0 to 10 on a Likert Scale) With Before and After Their Orthopedic Rotation

	Before Rotation (Mean)	After Rotation (Mean)	∆ = After − Before (Mean)	N
**Steps for tibia intramedullary nail**	**2.0**	**6.8**	**4.8**^a^	**5**
**Applying tension band to olecranon fracture**	**1.0**	**5.6**	**4.6**^a^	**5**
Applying a thumb spica splint	1.8	6.3	4.5^a^	6
**Inserting traction pin**	**3.8**	**8.0**	**4.2**^a^	**6**
Interpreting radiographs of tibia/fibula	3.0	7.0	4.0^a^	6
Applying a radial gutter splint	1.8	5.8	4.0^a^	6
Applying a coaptation splint	2.0	5.8	3.8^a^	6
When to use proximal tibia vs. distal femur traction	2.2	6.0	3.8^a^	6
Ordering imaging for a tibia fracture	2.2	6.0	3.8	6
**Classifying ankle fracture with Lauge Hansen**	**2.7**	**6.2**	**3.5**^a^	**20**
Classifying ankle fracture with Weber	4.5	7.9	3.4^a^	20
**Applying an ulnar gutter splint**	**3.0**	**6.0**	**3.0**^a^	**6**
Cannulated screws for femoral neck	3.7	6.7	3.0^a^	6
Classifying peds SCH fx	3.3	6.1	2.9^a^	7
Applying short leg splint	4.9	7.8	2.9^a^	20
Interpreting plain radiograph of ankle	3.8	6.5	2.7^a^	20
Ordering imaging for peds elbow injury	2.3	5.0	2.7^a^	7
**Interpreting peds elbow radiograph**	**2.4**	**5.0**	**2.6**^a^	**7**
Ordering imaging for adult elbow injury	2.8	5.4	2.6^a^	5
**Applying long arm splint**	**4.0**	**6.5**	**2.5**^a^	**24**
**Reducing an ankle fracture**	**3.2**	**5.7**	**2.5**^a^	**20**
Setting up skeletal traction	4.3	6.8	2.5^a^	6
Classifying intertrochanteric fracture	4.8	7.3	2.5^a^	6
Ordering imaging for hip fracture	3.2	5.6	2.4	5
Reducing distal radius fracture	3.6	5.9	2.3^a^	17
Performing intraarticular ankle block	2.6	4.9	2.3^a^	20
Hematoma block for distal radius fracture	3.1	5.3	2.2^a^	17
Interpreting plain radiograph of wrist	4.1	6.3	2.2^a^	17
Interpreting adult elbow radiograph	3.6	5.8	2.2^a^	5
Ordering imaging for ankle fracture	3.8	5.9	2.1^a^	20
Interpreting plain radiographs of the hip	4.3	6.3	2.0^a^	8
Ordering imaging for distal radius fracture	3.8	5.8	1.9^a^	17
Determining who needs skeletal traction	4.5	6.2	1.7^a^	6
**Classifying femoral neck fracture**	**4.9**	**6.3**	**1.4**^a^	**8**

N = number of student responses.

a Statistically significant (*P* < 0.05).

Topics shaded in bold are shown in Figure 1.

**Figure 3:**
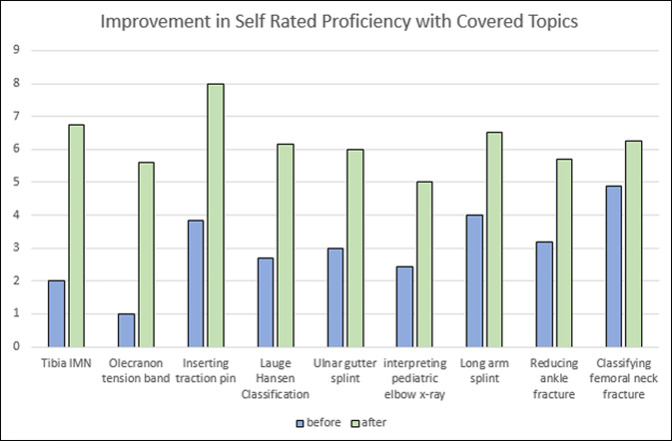
The chart illustrates the surgical skills listed in Table 2. Students responded with an improvement in comfort level with surgical skills.

At the end of each rotation, students were asked to fill out anonymous, voluntary evaluations. Thirty-two of 46 students (70%) returned their surveys. As a measure of improvement in self-rated proficiency with topics covered by the subintern curriculum, the students rated their comfort level with specific skills before and after their rotation, using a 0 to 10 Likert scale. Data analysis revealed that students reported notable improvement in comfort with covered fundamental orthopaedic skills. See Table 2 for a full list. Figure 3 illustrates this improvement for a selection of topics (shaded in grey in Table 1 for reference). The topics the students rated the most improvement in from before to after their rotation were steps for tibia intramedullary nail (∆ = 4.8, *P* < 0.05), applying a tension band wires and plates to an olecranon fracture (∆ = 4.6, *P* < 0.05), and applying a thumb spica splint (∆ = 4.5, *P* < 0.05).

Qualitative data review revealed themes that were important to the students. All students (n = 32) felt the time and effort put into their case presentations during week 4 of the subintern curriculum was beneficial. All students who filled out the surgical skills evaluation (n = 40) answered yes when asked if the surgical skills laboratory should be repeated for future rotating medical students and if the skills they learned would be helpful as they start residency (n = 40).

As part of the end of rotation evaluation form, students were asked which laboratory was the most beneficial. Eight of 32 responded that the week 1 surgical skills laboratory was the most beneficial. Six of 32 felt the tibia fracture laboratory with intramedullary nailing was the most beneficial. When asked which station of the orthopaedic surgical skills laboratory was the most helpful, 28 of 40 students chose the station with drilling/plates/screws; 14 students specifically mentioned lag screw insertion in their open-ended response. Tables 3 and 4 show a sample of the open-ended responses to the above questions.

**Table 3:** Students Answered What They Felt was the Most Helpful Surgical Skills Station

Lag screw and plate, rarely learn about hardware.
Knot tying using the rope, allowed you to see how the knots lay as you throw them.
Station with screws, we always hear “3.5” in the OR and I had no idea what that meant.
Drilling/screws, residents were great about breaking down the theory behind each step.
Lag screw, got to cut, reduce, drill, measure, put a screw in.
Drilling, sawing, screws because we are never given these tools.
Drill/screw, helps understand procedures in OR and reduction techniques.
Suturing, it's a skill we are expected to do as students and should be mastering.
Lag screw, new concept I hadn't learned about.
Plating station because it was brand new and explained the concepts I learned in videos.

**Table 4:** Students Answered Which Laboratory Session They Felt was the Most Beneficial

Laboratory with drilling, knots, etc. Helped clear up how and why we do things a certain way.
Hands on drilling, suturing, sawing. These are skills we have no experience with and often are not allowed to perform.
Tibial shaft fracture workshop, great to work hands on with chiefs, now know most steps for tibial nail.
Skills labs, practiced skills in less stressful conditions with maximal instruction.
Tibia fractures was awesome, lecture then sawbones great.
Surgical skills laboratory; getting comfortable with equipment; informal discussions with residents on biomechanics.
Enjoyed intramedullary nailing the most. Learn best when things are hands on.
Plating and screw insertion, good to learn basic principles behind what we do.
Splinting, don't get much teaching on this and it's important to learn before intern year.
Splinting, most relevant/something we can actively help with in the OR.

## Discussion

For fourth-year medical students, there are many benefits of completing orthopaedic subinternships. They provide the opportunity to experience multiple orthopaedic programs with different locations, teaching styles, and variations in program size/structure. This helps students refine characteristics that are important to them in their search for a residency program, where they will spend five challenging years in training. In addition, residency applications in orthopaedic surgery are competitive, and face-to-face time in a clinical environment is a chance for students to set themselves apart from the large pool of other applicants. A major purpose of orthopaedic sub-internships is to obtain a future residency position. In a survey by O'Donnel et al, students chose “desire to match at a program” as the most important factor in arranging an away rotation.^1^ In addition, program directors factor subinternship performance into the resident selection process.^4^ In fact, it has been shown that over half of orthopaedic applicants match at either their home program or at a program where they rotated.^2^

We feel that a subinternship should be more than an audition rotation. Because only 15% of American medical schools require musculoskeletal clinical rotations and only 35% of schools offer orthopaedics as an elective, orthopaedic applicants may be starting their sub-internships without any previous experience rotating in musculoskeletal subspecialties.^3^ By the time they start residency, graduating medical students are expected to meet Level 1 ACGME orthopaedic milestones. Petravick et al. showed that recent graduates who matched into orthopaedic residencies were only comfortable with half of the Level 1 milestones.^5^ Therefore, teaching during subinternships is especially important. Subinternships are an opportunity to educate future residents and to prepare them for -internship with targeted knowledge and skill development. To optimize the orthopaedic medical student rotation, Campell et al. suggested including weekly workshops and didactic sessions in addition to clinical teaching.^6^

Unfortunately, most surgical subinternships remain unstructured, lacking specific learning objectives and formal educational sessions. In a position study published in 2015, Issa et al.^7^ suggested that there is a gap which needs to be addressed in the transition between fourth year of medical school and the first year of surgical residency. A structured curriculum, with didactics and hands-on laboratory test results designed and taught to a senior medical student level, is an essential step to improve this transition. In a survey of fourth-year medical students applying to general surgery residency, only 21% of subinternships included any formal laboratories.^8^ To create a curriculum, Issa et al.^7^ proposed several domains including creating a sub-internship course director, establishing goals and objectives, and setting aside weekly protected time for educational activities. These can be applied to any specialty, including orthopaedic surgery.

The goal of the current educational initiative at our academic medical center was the creation of a new orthopaedic subinternship curriculum. The weekly curriculum has been enthusiastically received by participating students, who reported notable improvement in their comfort with covered topics after their rotations.

At this point, we hope to share and grow this program to include other orthopaedic institutions. The strengths of the curriculum are that it provides education to orthopaedic subinterns with learning objectives based on the ACGME milestones. The laboratory test results and didactics are designed and taught at a fourth-year medical student level. The laboratory environment provides a low-stakes setting. In addition. The curriculum offers teaching opportunities to residents, who design and run each of these sessions. The monthly topics can be standardized so that students who do more than one subinternship will not cover the same topics at different rotations. Because the number of residents and their enthusiasm for teaching differs between programs, recruiting is a potential anticipated challenge. Other possible challenges include finding an appropriate setting for the laboratory test results, setup, and access to materials including implants and sawbones. We found the most challenging aspect of running the curriculum was the creation of a comprehensive supply list. With experience, we were able to better plan ahead for the sessions and have all the essential tools and sets available and ready to go at the end of a busy clinical day. This detailed planning greatly aids the efficiency and success of these skill building sessions. Our institution is grateful to have access to an orthopaedic skills laboratory and to have the assistance of a laboratory coordinator to help with setup. In addition, our industry partner has been valuable in their enthusiasm to support education by providing teaching sets.

Several limitations are overserved in this study. The experience is limited to one academic institution, and no data are found from before the curriculum for comparison. In addition, as feedback was collected from students, changes were made to the content of the program and to the evaluation forms, limiting the number of possible student responses. The main limitation is that data to objectively evaluate the effectiveness of the curriculum was not available (for example, quizzes or timed tasks completed before versus after a teaching session). In addition, we do not have feedback from residents or attendings to confirm whether concepts learned in the curriculum translated to improvements in real-world performance.

In conclusion, this initiative was successful in designing and implementing a structured curriculum for orthopedic subinterns. The program featured didactic and skills sessions which were very well received by the student learners and resident teachers. We are hopeful that sharing our experience, including the highlights and challenges, with other orthopaedic educators and residency programs will enable them to consider implementing a similar program for their own orthopaedic subinternship students. In the future, we aim to help expand the program regionally and nationally to support the process of refocusing and increasing the overall educational value of the orthopaedic subinternship experience.
